# Limited Antibodies to Henipaviruses Detected Among Nigerian Horses Using Pseudotyped Virus Neutralization Tests

**DOI:** 10.3390/v18090961

**Published:** 2026-09-02

**Authors:** Kitty Donnelly, Meshach M. Maina, Ola Elbohy, Olaolu T. Olufemi, Edward Wright, Janet M. Daly

**Affiliations:** 1 One Virology, Wolfson Centre for Global Virus Research, School of Veterinary Medicine and Science, University of Nottingham, Sutton Bonington LE12 5RD, UK; svykd2@nottingham.ac.uk (K.D.); meshach.maina@nottingham.ac.uk (M.M.M.); ola.elbohy@biology.ox.ac.uk (O.E.); olufemit@unijos.edu.ng (O.T.O.); 2 School of Life Sciences, Faculty of Science, Engineering and Medicine, University of Sussex, Brighton BN1 9RH, UK; ew323@sussex.ac.uk

**Keywords:** pseudotype virus neutralization test, henipavirus, Nipah virus, seroprevalence

## Abstract

The Hendra virus (HeV; *Henipavirus hendraense*) and Nipah virus (NiV; *Henipavirus nipahense*), members of the genus *Henipavirus*, are high-consequence pathogens that have caused multiple zoonotic outbreaks with high mortality. Frequently, pigs or horses have acted as bridging hosts between bats, the reservoir hosts, and people. We used pseudotyped viruses with the fusion (F) and glycoprotein (G) envelope proteins of the NiV and Ghana virus (GhV; *Henipavirus ghanaense*) in neutralization tests (PVNTs) to screen serum samples from 138 apparently healthy horses in northwest Nigeria. No antibodies to GhV were detected in the 138 samples tested. Two serum samples were positive for antibodies to NiV on an initial screen using a single serum dilution, giving a prevalence of 1.45% (95% CI: 0.4–5.13%). Both positive samples were from female, non-local breeds of horses that were used for polo and were 15 and 20 years old. The only significant risk factor found through Chi-squared analysis was the older age category (*p* = 0.028). On titration in a PVNT, a half-maximal inhibitory concentration was calculated for both samples (IC_50_ = 11.6 and 16.8). These data suggest the limited exposure of horses to NiV in the sample region but confirms that horses can act as a sentinel population for the circulation of henipaviruses.

## 1. Introduction

*Henipavirus* is a genus in the family *Paramyxoviridae*, belonging to the *Orthoparamyxovirinae* subfamily [1]. Species of henipavirus include *Henipavirus hendraense*, *Henipavirus nipahense*, or *Henipavirus ghanaense*, commonly known as Hendra virus (HeV), Nipah virus (NiV), or Ghana virus (GhV). Nipah and henipaviral diseases are listed in the World Health Organization R&D Blueprint, which prioritizes diseases for research and development in emergency contexts [1]. The global number of confirmed cases remains relatively low, but the case fatality of HeV infections can range from 40% to 100%, depending on the strain and context [2]. The Hendra virus was the first *Henipavirus* species to be discovered in 1994. Initially thought to be an equine morbillivirus, the disease caused the death of 20 horses and a horse trainer in Brisbane, Australia. Since then, there have been 89 confirmed cases of HeV in horses and 4 people have died due to viral spillover [3], but HeV has not been found outside Australia.

The Nipah virus was identified during an investigation of encephalitis cases among pig farmers in Malaysia from 1998 to 1999. The outbreak, which spread to Singapore, resulted in 283 human cases and 109 fatalities [4]. As pigs were found to be the reservoir hosts, the Malaysian government culled one million pigs to control the outbreak. This cost the Malaysian government US$97 million in economic losses, but there have since been no further NiV outbreaks in Malaysia or Singapore [5]. Outbreaks in Bangladesh were first reported in 2001 and have since occurred almost annually in some areas of Bangladesh and India [6,7]. The overall case fatality of NiV throughout Southeast Asia (Bangladesh, India, Malaysia, Philippines, and Singapore) between its emergence and 2016 was estimated to be 61% [8]. The Nipah virus is primarily associated with fruit bats of the genus *Pteropus*, which are the reservoir hosts for multiple species of henipavirus [9]. These bats are endemic to tropical and subtropical regions of Asia, East Africa, and Australia. The spread of NiV occurs primarily through direct contact with infected bodily fluids like urine, feces, and saliva. Additionally, foodborne transmission has been documented, notably during the outbreaks in Bangladesh, which were linked with the consumption of date palm sap contaminated with bat secretions [10]. Although the transmission from bats to pigs facilitated subsequent human infections in Malaysia, horses acted as bridging hosts during an outbreak in the Philippines in 2014, where 17 human cases resulted in 81% mortality [11]. In a study conducted in Kaduna State, Nigeria, the seroprevalence determined using iELISA and samples from 200 horses was found to be 15.5% [12]. In a study published in 2022, 536 horses and 508 pigs were sampled across seven Northern Nigerian states [13]. This study reported a 0.56% prevalence of NiV antibodies in horses using Luminex binding assays, although these samples gave negative results when tested in iELISA and serum neutralization tests.

The Ghana virus was first identified in 2008 from fecal samples of *Eidolon helvum* fruit bats in Ghana [14]. Antibodies to GhV have also been detected in Eidolon bats in Madagascar, Ghana, and Cameroon [14,15,16]. The virus has only been isolated from bats thus far, but the spillover potential of GhV is being increasingly recognized, especially as bats are frequently butchered for meat across West Africa. A study in Cameroon indicated that henipavirus-seropositive individuals all had close contact with and were involved in butchering bats [16]. In a study of 304 bat serum samples from hunted *Eidolon helvum* bats in Nigeria, 29.6% (16/54) of the samples were positive for NiV and 66.7% (36/54) for GhV, with 13 samples positive for both viruses [17]. The potential for cross-neutralization and false positives was acknowledged as GhV antibodies have been shown to cross-neutralize NiV [16].

Understanding the prevalence and distribution of henipaviruses is crucial for developing effective surveillance, prevention, and control strategies, and ultimately reducing the risk of outbreaks and protecting public health. To date, the circulation of NiV has not been confirmed in Africa. However, as noted in a recent World Health Organization (WHO) Rapid Risk Assessment (RRA) evaluating the global public health risk posed by NiV, the detection of antibodies to henipaviruses in African bats indicates ecological suitability [18]. Equids are potentially informative sentinels that are more readily sampled than bats. Neutralization tests using pseudotyped viruses (PVs) with both the fusion (F) and glycoprotein (G) envelope proteins are often used in the serosurveillance of henipaviruses as they can be performed in lower-containment-level facilities than neutralization tests with authentic viruses [16,17,19,20,21]. In a study comparing the conventional plaque reduction neutralization test (PRNT) and PVNT, the specificity of the PVNT was 94–100% [21]. However, the PRNT was able to detect lower levels of antibodies compared to the PVNT.

The aim of the current study was to screen equine serum samples collected in northwest Nigeria for antibodies to both NiV and GhV using PVNTs.

## 2. Materials and Methods

### 2.1. Serum Samples

**Figure 1:**
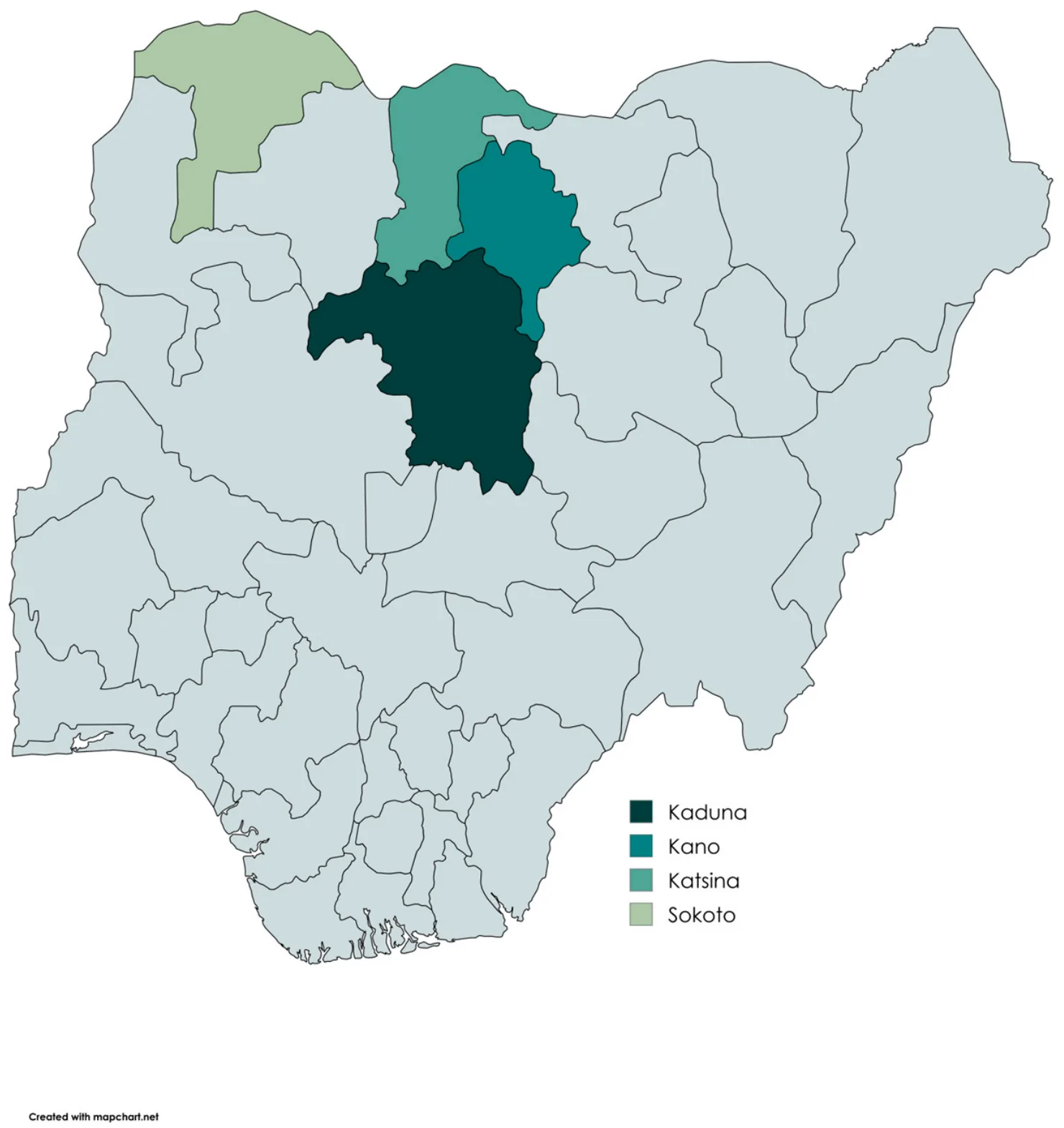
Map of Nigeria showing the four northwestern states from which equine serum samples were obtained: Kaduna, Kano, Katsina, and Sokoto.

Serum samples used in this project were a subset of samples of 138 taken from 830 apparently healthy horses in four states (Kaduna, Kano, Katsina, and Sokoto) of northwest Nigeria between 2019 and 2020 [22] (Figure 1).

Blood samples were taken by jugular venipuncture and the serum harvested and heat inactivated prior to shipping to the UK. Sample size was calculated using previous estimates of henipavirus seroprevalence in Nigerian equids [12,13] and the online EpiTools application [23], assuming a desired precision of ±0.05 and a 95% confidence level. The subset of samples was selected to ensure that different demographic factors were proportionately represented. These included location/state (Kano, Kaduna, Katsina, and Sokoto), age (young, adult, or old), different purposes (polo, traditional durbar, or mixed), different breeds (Arewa, Argentine, Sokoto, South Africa, or Talon), and sex (male/female).

Age categories were defined as young horses being between the ages of >3 months to <5 years, adults being 5 years to <15 years, and old horses being 15 years and above. The most common group of horses were adult males, which are commonly used in the traditional durbar celebrations (Supplementary Table S1). Associations between demographic factors and positivity were analyzed using Fisher’s exact test in GraphPad Prism (version 11.0.0).

### 2.2. Cells

HEK293T/17 cells (derived from human embryonic kidney) were used both to produce PVs and as target cells for virus neutralization tests. The cells were maintained in DMEM supplemented with 10% fetal bovine serum and 1% Gibco™ penicillin–streptomycin solution (10,000 U/mL penicillin and 10,000 μg/mL streptomycin; Fisher Scientific UK Ltd., Loughborough, UK).

### 2.3. Pseudotype Virus Production

The F and G envelope protein gene sequences of NiV Bangladesh strain (GenBank accession number JN808864.1) and GhV strain M74a (GenBank accession number HQ660129) were synthesized with human codon optimization. The NiV G was a kind gift from Prof. Lambe, University of Oxford, while the NiV F, GhV G, and F were synthesized by GeneArt. Each envelope protein gene was cloned into the pCAGGS expression plasmid to produce pCAGGS-FNiV, pCAGGS-GNiV, pCAGGS-FGhV, and pCAGGS-GGhV.

The methods for producing NiV and GhV PVs were independently optimized for cell seeding density, core plasmid, plasmid ratios, and length of incubation (see results). Briefly, the day before transfection, 2 mL HEK293T/17 cells were seeded in a 6-well plate. The optimized ratio of core plasmid (p8.91 or psPAX2), pCAGGS-F, pCAGGS-G, and pCSFLW was mixed in 100 μL Opti-MEM (Gibco™) in an Eppendorf tube. In a separate tube, 100 μL Opti-MEM was added to 7.5 μL Invitrogen™ Lipofectamine 2000 Transfection Reagent (Fisher Scientific UK Ltd., Loughborough, UK). Control constructs included a no-envelope (Δ-envelope) control, containing only the core and reporter without the glycoproteins, and a positive control, using 0.65 μg pVSV-G as an envelope protein instead of the F and G envelope protein plasmids. The two aliquots were mixed via brief centrifugation and left to stand for 30 min at RT. The media was replaced and 200 μL of each tube was added drop by drop. The plate was gently shaken and incubated at 37 °C, 5% CO_2_ for 24, 48, or 72 h. To harvest the PVs, 2 mL of the cell suspension was collected and filtered through a hydrophilic mixed cellulose ester (MCE) 0.45 μM membrane filter (Millex, Merck Life Sciences UK Limited, Gillingham, UK). The PVs were stored at −80 °C until use.

### 2.4. Titration of Pseudotyped Viruses

In the first column of a 96-well plate, PV was diluted to either 1:5 for GhV or 1:25 for NiV in 125 μL complete DMEM in quadruplicate. Then, 100 μL media (complete DMEM) was added to the remaining wells and 25 μL was mixed across the rows in a serial dilution, excluding column 12, which was used for the ‘cell control’. The plate was incubated at 37 °C for 1 h before 100 μL of 2 × 10^5^ HEK293T/17 cells/mL were added. The plate was centrifuged at 1200 rpm for 1 min, then incubated for 48 h at 37 °C, 5% CO_2_. After 48 h, the supernatant was gently removed from each well and replaced with 100 μL of a mix of 1:1 warmed DMEM and Steady Glo-substrate (Promega™, Chilworth, UK). Then, the plate was left for 10 min, and the wells were mixed with a pipette. The plate was read using a GloMax^®^ Discover Microplate Reader (Promega™) and the titer determined using a positive threshold of ≥3× the mean RLU/mL obtained for the cell control wells.

### 2.5. Pseudotyped Virus Neutralisation Tests

The WHO/BS/2023.2458 international standard for Nipah virus antibody [24] product number 22/130_NT (MHRA-NIBSC, South Mimms, UK) was used as a positive control in the NiV PVNT and to optimize the initial serum sample dilution. Donor horse serum (SR035C, Fisher Scientific UK Ltd., Loughborough, UK) was added to duplicate wells as a negative control serum.

For initial screening assays, each serum sample was diluted 1:80 with complete DMEM, and 100 μL per well was added to a 96-well clear-bottomed white cell culture plate (avoiding the outermost wells). Eight wells served as PV-only controls with 100 μL DMEM substituted for the serum. In the NiV PVNTs, two wells were used for the positive NiV control antibody diluted 1:80, whereas these wells were used for additional PV controls in the GhV PVNT as no positive control was available. Finally, the titrated PVs were diluted to around 1  ×  10^6^ RLU/mL and 100 μL was added to all wells except for four cell control wells. The mixture of PV and sera was incubated at 37 °C for 1 h before 50 μL of 2 × 10^5^ HEK293T/17 cells/mL was added to each active well. The plate was centrifuged at 1200 rpm for 1 min, then incubated at 37 °C, 5% CO_2_ for 48 h. Following incubation, the supernatant was gently removed, and 100 μL of a 1:1 mixture of Steady Glo-substrate (Promega) and DMEM was added to each well. The wells were left for 10 min, then mixed by a pipette, and the luciferase expression was read at a 0.5 s delay range using a GloMax^®^ Explorer Microplate Luminometer (Promega, Chilworth, UK). A sample was considered positive if the RLU/mL value met or exceeded the threshold defined by positive RLU/mL ≤ virus-only control RLU ÷ 2 (50% reduction).

To titrate the positive samples from the initial screening, the serum samples were serially titrated in two-fold dilutions from a starting dilution of 1:5. The NiV PV was added to each well at 1 ×  10^6^ RLU/mL. A normalized, variable-slope nonlinear curve was fitted and the IC_50_ of the sera calculated in GraphPad Prism (version 11.0.0).

## 3. Results

The optimization of the production of the NiV and GhV PVs resulted in the use of different cell-seeding densities and plasmid ratios (Table 1). There was no significant difference in titer using p8.91 or psPAX2 as the core plasmid and psPAX2 was chosen as the core plasmid for both NiV and GhV PVs. For both the NiV and GhV PVs, the media was harvested at 48 h, but, to maximize the titer of the GhV PV, the media was replaced and a second harvest taken at 72 h.

The titer of the GhV PV was 10^1.5^ lower than the titer of the NiV PV. Therefore, the screening PVNT was conducted with a lower-than-typical amount of PV so that the amount was equivalent for both. On the initial screening in the PVNT with a single serum dilution, no antibodies to GhV were detected in the 138 samples. Two serum samples were positive for antibodies to NiV, giving a prevalence of 1.45% (95% CI: 0.4–5.13%). Both positive samples were from ≥15-years-old Argentine breed mares that were used for polo. The only significant risk factor was the older age category (Table 2).

**Figure 2:**
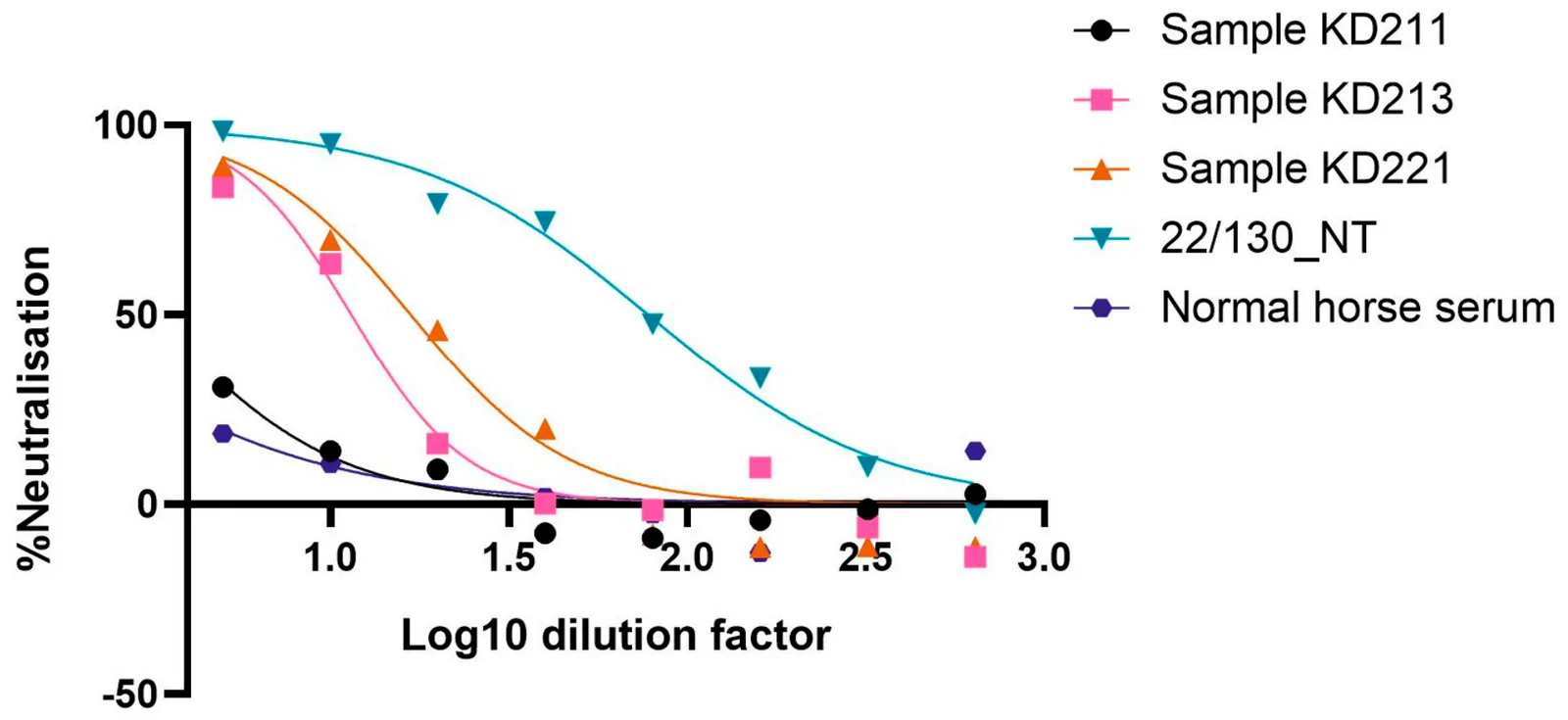
Normalized percentage neutralization values for the two equine serum samples that were positive in the screening pseudotyped virus neutralization test. Normalized, variable-slope nonlinear curves were fitted using constrained parameters for the logarithm of the serum dilution for samples KD211, KD213, and KD221; the 22/130 NT international reference standard serum served as a positive control and commercially available ‘normal horse serum’ as a negative control. A representative experiment with technical triplicates is shown.

The two samples (KD213 and KD221) that tested positive for NiV antibodies using a single dilution in the initial screening test were independently titrated on 293T/17 cells (Figure 2). IC_50_ values of 11.6 and 16.8 were obtained. The IC_50_ value calculated for the 22/130_NT reference serum was 77.6. Both commercially available ‘normal’ horse serum and a sample that tested negative in the initial screening (KD211) were confirmed to be negative on titration.

## 4. Discussion

Henipaviruses have become a cause of considerable epidemiological concern since their first emergence in 1994. Zoonotic transmission from bats frequently involves bridging hosts, specifically pigs and/or equids. In this study, we focused on the potential role of equids as a sentinel species for the detection of henipaviruses in northwest Nigeria. Second-generation lentivirus vectors have been successfully used to generate henipavirus PVs previously [19]. Henipavirus infection requires both the F and G envelope proteins, with a common ratio of 1:1 used, which was also applied to our pseudotypes after a series of optimization experiments. During the optimization of PV generation in this study, a marginally higher PV yield was obtained using psPAX2 compared to p8.91 to provide the core lentiviral proteins.

The Nipah virus is known to have the capacity to infect a wide range of mammalian cells [25]. Vero cells have been extensively used to study the authentic virus [25,26,27]; however, the presence of primate Trim 5α in African-green-monkey-derived Vero cells can restrict the retrovirus core of HIV-based PVs [28,29]. Although BHK cells were previously used as target cells for henipavirus PVs [17], we used HEK293T/17 cells as higher titers were obtained compared to BHK cells (unpublished data), likely because type I interferon production is limited in HEK293T cells [30].

To spare resources, serum samples were initially screened at a single dilution of 1:80, which was optimized for NiV using the 22/130 NT international reference standard serum. Cantoni et al. [17] similarly used a single point dilution of 1:100 for screening *Eidolon helvum* serum samples. Two samples were determined to be positive using a threshold of 50% reduction when compared to the signal obtained with the PV-only control wells, giving an overall prevalence of antibodies to NiV of 1.45% (95% CI: 0.4–5.13%). The titration of the positive samples resulted in the calculation of IC_50_ values of 11.6 and 16.8. As there are currently no commercial vaccines available to protect against henipaviruses in Africa, a demonstration of antibodies in horses suggests the natural exposure to the virus. Both samples were from older, female, polo horses sampled in Kaduna state. The age category (>15 years) was found to be the only statistically significant factor (*p* = 0.028). An older age is frequently associated with an increased risk of being positive for viral antibodies due to the greater opportunity for past exposure and repeated exposure, potentially boosting antibody levels so that they wane less rapidly. On the other hand, with only two positive samples from animals sharing the same demographic characteristics, it is not possible to rule out sex, breed, location, or purpose as confounding factors. Bats were observed roosting in trees adjacent to some stables during sampling; any future studies should record the proximity of bat roosts to sample sites and the species of bat.

In contrast to the seroprevalence of 1.45% we found, in a study conducted in Kaduna, the state from which positive samples were obtained in our study, the seroprevalence using iELISA was 15.5% among 200 horses [12]. However, a previous study in seven Nigerian states (only Kaduna overlapped with the current study) found a seroprevalence of only 0.56% in healthy horses using Luminex binding assays and the same samples gave negative results when subjected to serum neutralization tests using infectious virus [13]. The wide discrepancy in the seroprevalence between studies may reflect the use of different assays or the highly stochastic occurrence of exposure, as might be expected for rare interspecies transmission events. As our study only identified antibodies to NiV in 2 of 138 equine serum samples, further studies are required to draw any epidemiological inference on the circulation of henipaviruses among Nigerian horses.

No antibodies to GhV were detected, which was unsurprising as GhV has only been isolated from bats thus far. The lower titer of the GhV PV led to the use of a lower-than-typical amount of PV in the neutralization test, which might be expected to increase the sensitivity of the assay. However, no positive control serum was available to confirm the GhV PVs were correctly presenting the envelope proteins. The GhV PV could be used to generate a polyclonal antiserum to be used as a positive control and to optimize the PVNT sensitivity. The lower titer of the GhV PV may be because the surface expression of the GhV G protein is lower than that of the NiV G protein as most of the protein is retained in the endoplasmic reticulum [31]. A potential solution to improve the titer of PVs is to modify the foreign glycoprotein(s), including by exchanging the transmembrane domain and cytoplasmic tail with another protein. However, it has been determined that the cytoplasmic tail of the GhV G protein is responsible for the reduced formation of syncytia by the F protein, which is also restricted to bat cell lines. Hence, modifying either protein to enhance the PV titer could alter its biological properties.

The IC_50_ values of 11.6 and 16.8 obtained in our PVNT were moderate compared to 77.6 obtained for the 22/130_NT reference serum, which is from a pool of 36 people that had recovered from NiV infection. These lower values could reflect the timing of virus exposure (for example, antibodies could have waned over time). Alternatively, cross-neutralization between NiV and GhV antibodies has been described [16] and the low IC_50_ values could be ascribed to the measurement of only the cross-reactive component of an antibody response to another henipavirus species. A study conducted in Benue state, which is in the central region of Nigeria, investigated the presence of NiV and GhV antibodies in *E. helvum* bat populations using PVNT [17]. They found a prevalence of 29.63% for NiV and 66.7% GhV, with 13 of 54 samples (24%) being positive for both, suggesting that most of the positive findings for NiV could be due to the detection of cross-reactive antibodies to GhV.

Human cases of encephalitis in Africa of unknown etiology are often misattributed to malaria [32]. To ensure that any introduction or spillover is identified rapidly, the WHO recommends that NiV is included in the surveillance for acute encephalitis and severe respiratory illness [18]. Overall, using PVs allows the presence of neutralizing antibodies to be confirmed without the need for high levels of containment. Given that both NiV and GhV were only relatively recently discovered in Asia and Africa, respectively, but generate cross-reactive antibodies, it is possible that other as-yet-undiscovered henipavirus species circulate in Nigerian bats, as suggested by Adamu et al. [13]. If further species in the *Henipavirus* genus are discovered, PVs for antibody screening can be established rapidly based on the F and G protein sequences. Despite the low prevalence of NiV antibodies, monitoring the viral circulation in bridging host populations, such as horses, remains important due to the potential for viral mutations, spread into new ecological territories, and spillover into humans.

## Figures and Tables

**Table 1:** Optimization of conditions for producing Nipah virus (NiV) and Ghana virus (GhV) pseudotyped viruses.

Condition	NiV	GhV
Cell seeding density (per ml)	1 × 10^6^	8 × 10^5^
Core plasmid	psPAX2	psPAX2
Plasmid ratio *	1:0.5:0.5:1	1:1:1:1.5
Incubation time	48 h	48 & 72 h

* ratios of core:F:G:reporter plasmids of 1:1:1:1.5, 4:4:4:1, and 1:0.5:0.5:1 were tested.

**Table 2:** Fisher’s exact test analysis of risk factors associated with antibodies to Nipah virus.

Factor	N	Positive	*p* Value
AGE			
Young (<5 years)	47	0	**0.03**
Adult (5–14 years)	65	0	
Old (≥15 years)	26	2	
SEX			
Male	70	0	0.24
Female	68	2	
BREED ^1^			
Arewa	40	0	0.06
Argentine	25	2	
Sudan	23	0	
Talon	49	0	
STATE			
Kaduna	49	2	0.54
Kano	30	0	
Katsina	28	0	
Sokoto	31	0	
PURPOSE			
Mixed	31	0	0.73
Polo	69	2	
Traditional Durbar	38	0	
TOTAL	138	2	

N, number of samples in subset screened for antibodies to henipaviruses. ^1^ One sample was omitted from the analysis of breed as a risk factor because there was only a single ‘South African’ breed horse. Bold typeface indicates a significant *p* value (≤0.05).

## Data Availability

Data supporting the reported results can be found in the Supplementary Materials.

## Supplementary Materials

The following supporting information can be downloaded at: https://www.mdpi.com/article/10.3390/v18090961/s1.

## Abbreviations

The following abbreviations are used in this manuscript:

GhV	Ghanaian henipavirus
IC_50_	Half-maximal inhibitory concentration
NiV	Nipah virus
PV	Pseudotyped virus
PVNT	Pseudotyped virus neutralization test
RLUs	Relative light units
