# Supplementary material for: Limited Antibodies to Henipaviruses Detected Among Nigerian Horses Using Pseudotyped Virus Neutralization Tests
Source: Viruses. 2026 Sep 2;18(9):961. doi: 10.3390/v18090961 (PMC13612092; doi:10.3390/v18090961)
Supplement: Supplementary file 1 [file viruses-18-00961-s001.zip › viruses-4473475-supplementary.pdf]

Table S1

Sample No.	State	Age*	Age category	SEX	Breed	Purpose
SK138	SOKOTO	1.5	YOUNG	MALE	TALON	MIXED
SK133	SOKOTO	1	YOUNG	FEMALE	AREWA	MIXED
SK130	SOKOTO	7	ADULT	FEMALE	TALON	MIXED
SK122	SOKOTO	15	OLD	FEMALE	TALON	MIXED
SK121	SOKOTO	4	YOUNG	FEMALE	AREWA	MIXED
SK120	SOKOTO	6	ADULT	FEMALE	TALON	MIXED
SK113	SOKOTO	5	ADULT	MALE	AREWA	TRADITIONAL DURBAR
SK111	SOKOTO	15	OLD	MALE	TALON	TRADITIONAL DURBAR
SK109	SOKOTO	6	ADULT	MALE	AREWA	TRADITIONAL DURBAR
SK105	SOKOTO	8	ADULT	MALE	AREWA	TRADITIONAL DURBAR
SK101	SOKOTO	4	YOUNG	MALE	TALON	MIXED
SK96	SOKOTO	1	YOUNG	FEMALE	TALON	MIXED
SK90	SOKOTO	1	YOUNG	FEMALE	TALON	MIXED
SK88	SOKOTO	1	YOUNG	MALE	TALON	MIXED
SK85	SOKOTO	2	YOUNG	FEMALE	TALON	MIXED
SK82	SOKOTO	9	ADULT	MALE	AREWA	MIXED
SK61	SOKOTO	5	ADULT	MALE	AREWA	TRADITIONAL DURBAR
SK60	SOKOTO	7	ADULT	MALE	AREWA	TRADITIONAL DURBAR
SK55	SOKOTO	8	ADULT	FEMALE	SUDAN	MIXED
SK51	SOKOTO	5	ADULT	FEMALE	SUDAN	MIXED
SK48	SOKOTO	8	ADULT	FEMALE	SUDAN	POLO
SK47	SOKOTO	9	ADULT	FEMALE	SUDAN	POLO
SK45	SOKOTO	10	ADULT	MALE	TALON	MIXED
SK41	SOKOTO	10	ADULT	FEMALE	AREWA	MIXED
SK37	SOKOTO	5	ADULT	FEMALE	TALON	MIXED
SK35	SOKOTO	4	YOUNG	FEMALE	TALON	MIXED
SK24	SOKOTO	2	YOUNG	FEMALE	TALON	MIXED
SK21	SOKOTO	3	YOUNG	MALE	TALON	MIXED
SK20	SOKOTO	2	YOUNG	MALE	TALON	MIXED
SK17	SOKOTO	7	ADULT	MALE	TALON	MIXED
SK3	SOKOTO	4.5	YOUNG	MALE	TALON	MIXED
KT120	KATSINA	9	ADULT	FEMALE	TALON	POLO
KT111	KATSINA	4 months	YOUNG	FEMALE	SUDAN	POLO
KT107	KATSINA	12	ADULT	FEMALE	TALON	TRADITIONAL DURBAR
KT106	KATSINA	14	ADULT	FEMALE	ARGENTINE	TRADITIONAL DURBAR
KT102	KATSINA	12	ADULT	MALE	TALON	TRADITIONAL DURBAR
KT99	KATSINA	5	ADULT	FEMALE	SUDAN	TRADITIONAL DURBAR
KT97	KATSINA	9	ADULT	FEMALE	TALON	TRADITIONAL DURBAR
KT80	KATSINA	13	ADULT	MALE	AREWA	TRADITIONAL DURBAR
KT73	KATSINA	8	ADULT	MALE	AREWA	MIXED
KT62	KATSINA	9	ADULT	FEMALE	SUDAN	POLO
KT53	KATSINA	10	ADULT	FEMALE	AREWA	POLO
KT42	KATSINA	4	YOUNG	MALE	AREWA	MIXED
KT39	KATSINA	3	YOUNG	MALE	AREWA	MIXED
KT33	KATSINA	3	YOUNG	MALE	TALON	TRADITIONAL DURBAR
KT29	KATSINA	4	YOUNG	MALE	AREWA	MIXED
KT28	KATSINA	8	ADULT	MALE	TALON	TRADITIONAL DURBAR
KT26	KATSINA	4	YOUNG	MALE	AREWA	MIXED
KT24	KATSINA	1.5	YOUNG	MALE	TALON	MIXED
KT22	KATSINA	1	YOUNG	MALE	AREWA	TRADITIONAL DURBAR
KT21	KATSINA	8	ADULT	MALE	AREWA	TRADITIONAL DURBAR
KT19	KATSINA	8	ADULT	FEMALE	TALON	POLO
KT17	KATSINA	2	YOUNG	MALE	TALON	POLO
KT16	KATSINA	13	ADULT	FEMALE	TALON	POLO
KT13	KATSINA	15	OLD	MALE	TALON	POLO
KT12	KATSINA	11	ADULT	FEMALE	TALON	POLO
KT8	KATSINA	7	ADULT	FEMALE	TALON	POLO
KT4	KATSINA	8	ADULT	MALE	TALON	POLO
KT2	KATSINA	9	ADULT	MALE	TALON	POLO
KN310	KANO	4	YOUNG	FEMALE	AREWA	MIXED
KN300	KANO	3	YOUNG	FEMALE	AREWA	MIXED
KN261	KANO	9	ADULT	MALE	TALON	TRADITIONAL DURBAR
KN232	KANO	4	YOUNG	MALE	AREWA	TRADITIONAL DURBAR
KN210	KANO	8	ADULT	FEMALE	TALON	TRADITIONAL DURBAR
KN209	KANO	10	ADULT	FEMALE	AREWA	TRADITIONAL DURBAR
KN196	KANO	7	ADULT	MALE	AREWA	TRADITIONAL DURBAR
KN194	KANO	8	ADULT	MALE	AREWA	TRADITIONAL DURBAR
KN190	KANO	9	ADULT	MALE	AREWA	TRADITIONAL DURBAR
KN159	KANO	3	YOUNG	MALE	AREWA	TRADITIONAL DURBAR
KN136	KANO	11	ADULT	MALE	SUDAN	TRADITIONAL DURBAR

KN123	KANO	9	ADULT	MALE	AREWA	TRADITIONAL DURBAR
KN104	KANO	3	YOUNG	MALE	AREWA	TRADITIONAL DURBAR
KN98	KANO	12	ADULT	FEMALE	TALON	TRADITIONAL DURBAR
KN90	KANO	2	YOUNG	MALE	AREWA	TRADITIONAL DURBAR
KN79	KANO	7	ADULT	FEMALE	TALON	POLO
KN76	KANO	3	YOUNG	MALE	TALON	POLO
KN73	KANO	3	YOUNG	MALE	TALON	POLO
KN72	KANO	3	YOUNG	FEMALE	SUDAN	POLO
KN70	KANO	9	ADULT	FEMALE	TALON	POLO
KN66	KANO	7	ADULT	FEMALE	SUDAN	POLO
KN63	KANO	11	ADULT	FEMALE	SUDAN	POLO
KN61	KANO	4	YOUNG	FEMALE	AREWA	POLO
KN48	KANO	4	YOUNG	FEMALE	AREWA	POLO
KN47	KANO	6	ADULT	FEMALE	AREWA	POLO
KN40	KANO	3	YOUNG	FEMALE	SUDAN	POLO
KN43	KANO	8	ADULT	FEMALE	SUDAN	POLO
KN21	KANO	7	ADULT	MALE	AREWA	TRADITIONAL DURBAR
KN20	KANO	15	OLD	MALE	AREWA	TRADITIONAL DURBAR
KN7	KANO	10	ADULT	MALE	AREWA	TRADITIONAL DURBAR
KD254	KADUNA	4	YOUNG	FEMALE	SUDAN	POLO
KD250	KADUNA	30	OLD	MALE	ARGENTINE	POLO
KD246	KADUNA	15	OLD	FEMALE	ARGENTINE	POLO
KD245	KADUNA	18	OLD	MALE	ARGENTINE	POLO
KD239	KADUNA	25	OLD	MALE	ARGENTINE	POLO
KD234	KADUNA	18	OLD	FEMALE	ARGENTINE	POLO
KD229	KADUNA	18	OLD	FEMALE	ARGENTINE	POLO
KD224	KADUNA	10	ADULT	FEMALE	ARGENTINE	POLO
KD221	KADUNA	20	OLD	FEMALE	ARGENTINE	POLO
KD213	KADUNA	15	OLD	FEMALE	ARGENTINE	POLO
KD211	KADUNA	15	OLD	MALE	ARGENTINE	POLO
KD204	KADUNA	11	ADULT	FEMALE	SUDAN	POLO
KD196	KADUNA	17	OLD	MALE	SOUTH AFRICA	POLO
KD192	KADUNA	15	OLD	MALE	ARGENTINE	POLO
KD190	KADUNA	15	OLD	MALE	ARGENTINE	POLO
KD189	KADUNA	18	OLD	FEMALE	ARGENTINE	POLO
KD178	KADUNA	11	ADULT	FEMALE	ARGENTINE	POLO
KD177	KADUNA	20	OLD	FEMALE	ARGENTINE	POLO
KD175	KADUNA	25	OLD	FEMALE	ARGENTINE	POLO
KD164	KADUNA	15	OLD	FEMALE	ARGENTINE	POLO
KD162	KADUNA	4 months	YOUNG	FEMALE	ARGENTINE	POLO
KD158	KADUNA	16	OLD	MALE	ARGENTINE	POLO
KD157	KADUNA	25	OLD	MALE	ARGENTINE	POLO
KD156	KADUNA	30	OLD	MALE	ARGENTINE	POLO
KD153	KADUNA	20	OLD	FEMALE	ARGENTINE	POLO
KD149	KADUNA	15	OLD	FEMALE	ARGENTINE	POLO
KD139	KADUNA	4	YOUNG	MALE	AREWA	TRADITIONAL DURBAR
KD133	KADUNA	4	YOUNG	MALE	AREWA	TRADITIONAL DURBAR
KD124	KADUNA	2	YOUNG	MALE	AREWA	TRADITIONAL DURBAR
KD122	KADUNA	4	YOUNG	MALE	AREWA	TRADITIONAL DURBAR
KD120	KADUNA	9	ADULT	MALE	AREWA	TRADITIONAL DURBAR
KD118	KADUNA	1.5	YOUNG	MALE	TALON	POLO
KD117	KADUNA	2	YOUNG	MALE	TALON	POLO
KD116	KADUNA	2	YOUNG	FEMALE	TALON	POLO
KD103	KADUNA	12	ADULT	MALE	TALON	POLO
KD101	KADUNA	1	YOUNG	FEMALE	TALON	POLO
KD96	KADUNA	10	ADULT	FEMALE	SUDAN	POLO
KD93	KADUNA	6	ADULT	MALE	SUDAN	POLO
KD85	KADUNA	2	YOUNG	FEMALE	SUDAN	POLO
KD83	KADUNA	16	OLD	MALE	SUDAN	POLO
KD60	KADUNA	10	ADULT	MALE	SUDAN	POLO
KD44	KADUNA	7	ADULT	FEMALE	TALON	POLO
KD36	KADUNA	10	ADULT	MALE	TALON	POLO
KD35	KADUNA	8 months	YOUNG	FEMALE	SUDAN	POLO
KD31	KADUNA	5	ADULT	FEMALE	TALON	POLO
KD28	KADUNA	10	ADULT	MALE	SUDAN	POLO
KD16	KADUNA	13	ADULT	MALE	ARGENTINE	POLO
KD6	KADUNA	1	YOUNG	FEMALE	TALON	POLO
KD4	KADUNA	6	ADULT	FEMALE	SUDAN	POLO

*age in years except where otherwise stated
